# Endowing improved osteogenic activities with collagen membrane by incorporating biocompatible iron oxide nanoparticles

**DOI:** 10.3389/fbioe.2023.1259904

**Published:** 2023-10-12

**Authors:** Zheng Li, Yunyang Zhang, Qing Ye, Lei Wang, Hui Chen, Chenhui Xu, Peng Wang, Jianfei Sun

**Affiliations:** ^1^ State Key Laboratory of Bioelectronics, Jiangsu Key Laboratory for Biomaterials and Devices, School of Biological Science and Medical Engineering, Southeast University, Nanjing, China; ^2^ Center of Modem Analysis, Nanjing University, Nanjing, China; ^3^ Zhongda Hospital of Southeast University, Nanjing, China; ^4^ State Key Laboratory of Pharmaceutical Biotechnology, Department of Sports Medicine and Adult Reconstructive Surgery, Nanjing Drum Tower Hospital, The Affiliated Hospital of Nanjing University Medical School, Nanjing, China

**Keywords:** bone tissue regeneration, collagen membrane, iron oxide nanoparticles, osteogenic differentiation, Wnt/β-catenin signaling pathway

## Abstract

**Introduction:** Collagen-based scaffolds, renowned for their exceptional biocompatibility, have garnered attention as promising scaffolds for advancing bone tissue regeneration. Nevertheless, these scaffolds possess inherent limitations, such as notably compromised osteo-conductivity and osteo-inductivity.

**Methods:** Our study focused on enhancing the mechanical properties and osteogenic bioactivities of bovine-derived collagen membranes (CMs) from the Achilles tendon by incorporating FDA-approved iron oxide nanoparticles (IONPs), termed as IONP-CM. Three types of IONP-CMs (IONP-CM-0.5, IONP-CM-1, and IONPCM-1.5) were constructed by altering the amounts of feeding IONPs.

**Results:** Surface topography analysis demonstrated comparable characteristics between the IONP-CM and neat CM, with the former exhibiting augmented mechanical properties. *In vitro* evaluations revealed the remarkable biocompatibility of IONP-CMs toward mouse calvarial pre-osteoblast MC3T3-E1 cells, concurrently stimulating osteogenic differentiation. Mechanistic investigations unveiled that the osteogenic differentiation induced by IONP-CMs stemmed from the activation of the Wnt/β-catenin signaling pathway. Furthermore, *in vivo* bone regeneration assessment was performed by implanting IONP-CMs into the radial defect in rabbits. Results derived from micro-computed tomography and histological analyses unequivocally substantiated the capacity of IONP-CMs to expedite bone repair processes.

**Discussion:** IONP-CMs emerged as scaffolds boasting exceptional biocompatibility and enhanced osteogenic properties, positioning them as promising candidates for facilitating bone tissue regeneration.

## 1 Introduction

Collagens are the most abundant proteins that are widely distributed in various tissues such as skin, bone tendons, and ligaments. Collagen membranes (CMs) were considered scaffolds for bone tissue regeneration due to their excellent biocompatibility, low antigenicity, high swelling ability, facilitation of cell attachment and growth, and capacity to provide a favorable environment for the formation of new bone tissue (Sorushanova et al., 2019; Mathew-Steiner et al., 2021; Geahchan et al., 2022). Recently, there has been accumulating evidence that CMs can be used to fix and cover bone defect areas, protecting them from external interference and aiding in the restoration of bone shape and structure (Karamanos et al., 2021). CMs can also interact with the transplanted bone grafts, providing support and guidance for regeneration of bone tissue (Park et al., 2023). In fracture healing, CMs can be used to stabilize the fractured site and maintain alignment of the fractured ends, serving as an important adjunct and providing a suitable growth environment for facilitating the regeneration and repair of bone tissue (Wang et al., 2018). One advantage of the CM is its excellent plasticity to be cut and shaped according to specific demands to adapt to various bone defect morphologies (Karsdal et al., 2017). However, when used as repair scaffolds for bone tissue engineering (BTE), neat collagen still has some limitations such as poor mechanical strength and relatively low osteo-inductivity. Therefore, seeking methods for preparing collagen-based multifunctional scaffolds with enhanced mechanical strength and osteogenic bioactivities for BTE is highly desirable.

Recently, numerous nanomaterials, including gold nanomaterials, iron oxide nanoparticles (IONPs), silica nanoparticles, and magnesium oxide nanoparticles, have garnered significant interest within the realm of bone tissue regeneration due to their customizable physicochemical properties, excellent biocompatibility, and ability to manipulate the fate of biological cells (Qi et al., 2019; Zheng et al., 2022). Among them, IONPs have emerged as compelling agents, extensively harnessed as osteogenic bioactive factors to foster the osteogenic differentiation of biological cells (Wang et al., 2017; Yu et al., 2020). Particularly, PSC-coated IONPs (γ-Fe_2_O_3_), known by the trade name ferumoxytol, stand out as the only inorganic nanodrug sanctioned by the Food and Drug Administration (FDA) for addressing iron deficiency anemia, revealing the excellent biocompatibility and potential clinical translation of IONPs (Kaittanis et al., 2014). Previous studies demonstrated the capacity of PSC-coated IONPs to augment the osteogenic differentiation of human bone-derived mesenchymal stem cells (hBMSCs) via activation of the mitogen-activated protein kinase (MAPK) signaling pathway (Wang et al., 2016). Moreover, the incorporation of IONPs has the potential to empower existing bone repair scaffolds with elevated osteogenic potency. Chen et al. reported the layer-by-layer assembly of IONPs on the surface of 3D fibrous PLGA/PCL scaffolds fabricated by the electrospinning technique. The osteogenic differentiation of adipose-derived stem cells was enhanced more than twofold through the magnetic properties of IONPs (Chen et al., 2018). Moreover, introducing IONPs could enhance the mechanical properties of bone repair scaffolds (Hu et al., 2021). Hence, incorporating biocompatible IONPs into a bovine-derived CM (IONP-CM) might endow it with improved mechanical properties and osteogenic bioactivity to promote the outcomes of BTE strategies.

**SCHEME 1:**
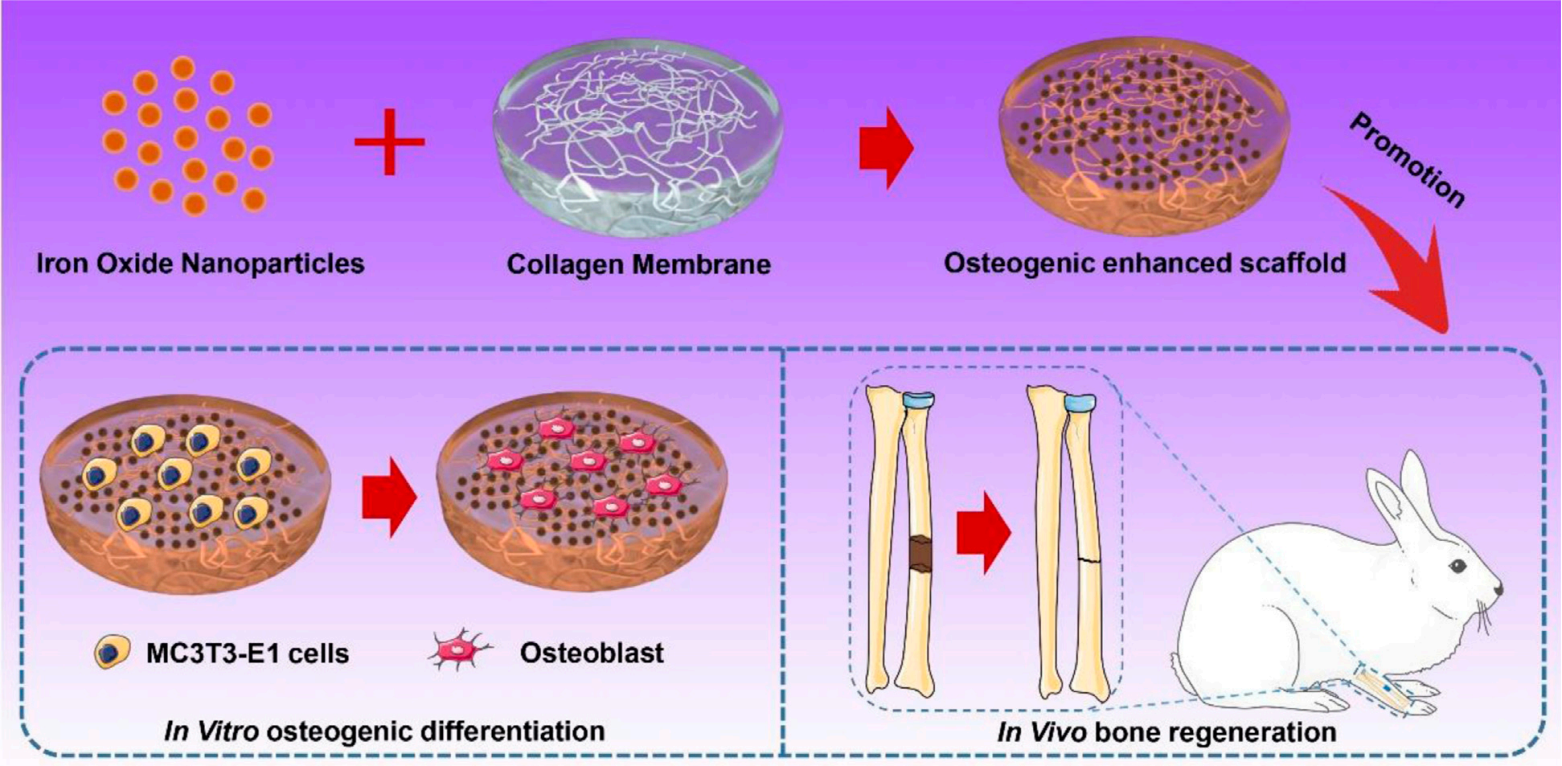
Schematic of the preparation of IONP-CMs for *in vitro* osteogenic differentiation and *in vivo* bone regeneration.

This study prepared an IONP-incorporated CM-based repair scaffold for bone tissue regeneration. Fundamental properties, including morphological, surface chemical, and mechanical features, of IONP-CMs were assessed using field-emission scanning electron microscopy (FE-SEM), atomic force microscopy (AFM), Fourier transform infrared spectroscopy (FTIR), and mechanical testing. In *in vitro* investigations, the biocompatibilities of IONP-CMs were evaluated using the cell viability assay. Moreover, their osteogenic properties were evaluated through the analysis of alkaline phosphatase (ALP) activity, mineralized nodule formation, and the expression of osteogenesis-related genes in the mouse calvarial pre-osteoblast cell line MC3T3-E1. IONP-CM-mediated osteogenesis was mechanistically investigated. *In vivo* bone regeneration performance was assessed using a radial defect model of rabbit. We hypothesized that biocompatible IONP-CMs with enhanced osteogenic activity and mechanical properties might be utilized as potential scaffolds for bone tissue regeneration (Scheme 1).

## 2 Materials and methods

### 2.1 Preparation of IONP-CMs

The bovine-derived CM was extracted from the bovine Achilles tendon using a previously reported procedure (Al-Maawi et al., 2018). Briefly, an adult bovine Achilles tendon without sarcolemma and fascia was washed with normal saline 8 times, immersed in alcohol solution (75%) for 15 min, and frozen at −60°C for 20 min. After that, slices were obtained by cutting at a thickness of 0.6 mm, followed by immersing in the acetic acid solution for 1 h and washing with phosphate-buffered saline (PBS) and distilled water 20 times. Subsequently, trypsin digestion was carried out, followed by dispersion through probe sonication at 80% of the maximum amplitude for 40 h and washing with PBS and distilled water 20 times to obtain an acellular dermal matrix (ADM). The ADM was immersed in formaldehyde solution for 2 h and washed with PBS and distilled water 20 times to obtain the ADM CM. Finally, PSC-modified IONPs (obtained from Jiangsu Key Laboratory for Biomaterials and Devices) were mixed with the ADM CM at 35°C for 15 min (final Fe concentrations were 0.5, 1, and 1.5 mg/mL). The IONP-CM was lyophilized, sterilized using cobalt-60, and stored at 4°C until further use.

### 2.2 Characterization of IONP-CM

The morphologies of the as-prepared IONP-CM were observed under an AFM (Signal Hill, CA, United States of America) and a field-emission SEM (Zeiss Supra 40 Gemini, Germany). The mechanical characteristics of the prepared IONP-CM were assessed using a universal material testing machine (Shimadzu Co., Japan). The rheological features of the IONP-CM were determined using a modular advanced rheometer system (HAAKE MARS, Thermo Fisher Scientific, United States of America). This involved conducting strain–sweep measurements over a range of 0.01%–10% strain amplitude at a frequency of 6.28 rad/s, as well as frequency–sweep measurements at frequencies ranging from 1 to 30 rad/s with a fixed 1% strain amplitude. For the IONP release test, the IONP-loaded CM scaffolds were precisely shaped into uniform squares (10 mm × 10 mm). After that, they were co-incubated with 1.5 mL of PBS at 37°C. The concentration of Fe ions released from the scaffolds was determined at predetermined time intervals spanning a 7-day period using inductively coupled plasma atomic emission spectrometry (ICP-OES, Agilent 5800, United States).

### 2.3 Cell culture

MC3T3-E1 cells were employed for cell culture experiments. The cells were maintained in a growth medium comprising α-MEM (Gibco, United States), 10% (v/v) fetal bovine serum, and 1% (v/v) penicillin/streptomycin (Gibco) and cultured at 37°C in a 5% CO_2_ atmosphere. For inducing osteogenesis, MC3T3-E1 cells were incubated in an osteogenic differentiation medium, containing a growth medium supplemented with 0.1 μM dexamethasone (Gibco), 50 μg/mL ascorbic acid (Gibco), and 10 mM β-glycerophosphate (Gibco), at 37°C in a 5% CO_2_ atmosphere.

### 2.4 Cell viability assessment

MC3T3-E1 cells were meticulously distributed into 96-well plates (5 × 10^3^ cells/well) and cultured for 24 h. Subsequently, the cells were subjected to immersion in a growth medium that was enriched with the leaching solution obtained from the IONP-CMs. This leaching solution harbored distinct iron concentrations set at 0.5, 1, and 1.5 mg/mL, thereby generating the designated formulations: IONP-CM-0.5, IONP-CM-1, and IONP-CM-1.5. Following the periods of 1, 3, 5, and 7 days of incubation, the cells underwent a thorough washing regimen with PBS. Subsequently, 10 μL of CCK-8 reagent was added to each well. Following incubation of 1 h at 37°C, the resultant reaction was quantified through assessment on a microplate reader (Multiskan GO, Thermo Fisher Scientific) at an optical wavelength of 450 nm, thereby providing a precise determination of cell viability.

### 2.5 Live/dead staining assay

IONP-CM-0.5, IONP-CM-1, and IONP-CM-1.5 were crafted into cubic geometries (10 mm × 10 mm). These samples were subjected to a rigorous sterilization procedure involving ultraviolet irradiation for 24 h. Following this, the sterilized samples were deftly transferred to 24-well plates, setting the stage for subsequent cellular assessment. MC3T3-E1 cells were seeded onto the surface of the IONP-CM samples (1 × 10^3^ cells square centimeter). Following a day of incubation, the cells were washed with PBS three times. Subsequently, the cells were stained using Calcein-AM/PI. The cells were subsequently scrutinized using a laser scanning confocal microscope (Olympus 141 FV3000, Tokyo, Japan).

### 2.6 Cell adhesion

The cell adhesion assays were conducted employing phalloidin/DAPI staining as previously described (Huang et al., 2023). MC3T3-E1 cells were cultured onto IONP-CMs treated as earlier for 2 h. Subsequently, the cells were subjected to rigorous washing with PBS three times. Following this, a fixation step was carried out, involving the use of a 4% paraformaldehyde solution for 10 min and treatment with 0.1% Triton X-100 for effectively facilitating permeabilization. Consecutively, the cells were subjected to a dual-staining regimen employing phalloidin and DAPI. The cells were then observed using a laser scanning confocal microscope (Olympus 141 FV3000), thus providing a detailed insight into the dynamics of cell adhesion.

### 2.7 ALP and ARS staining assays

MC3T3-E1 cells were cultured in 12-well plates at a density of 3 × 10^4^ cells/well at 37°C in 5% CO_2_; the culture medium was a growth medium supplemented with the leaching liquor of IONP-CMs. For ALP staining, the cells were cultured for 14 days, washed with PBS three times, fixed with 4% paraformaldehyde for 10 min, and stained using the BCIP/NBT ALP Color Development Kit. For ARS staining, the cells were cultured for 21 days and subsequently stained with 5% Alizarin Red S staining solution. The stained plates were observed and photographed using an inverted optical microscope (Olympus IMT-2, Tokyo, Japan).

### 2.8 Real-time PCR

Total RNA was extracted from the MC3T3-E1 cells that had undergone treatment with the leaching solution obtained from IONP-CMs for 7 and 14 days using the RNA-Quick Purification Kit (Yishan Biotech, Shanghai, China). Subsequently, cDNA was obtained, followed by meticulous analysis using the HiScript IIQ RT SuperMix for quantitative polymerase chain reaction (qPCR) (Vazyme Biotech, Nanjing, China). The qPCR amplification was performed using the ChamQ SYBR Color qPCR Master Mix, procured from Vazyme Biotech. The qPCR reactions were carried out using the ChamQ SYBR Color qPCR Master Mix, with the gene-specific primers outlined in Supplementary Table S1. Notably, the quantification of target transcripts was subjected to normalization, with reference to the levels of internal reference transcripts, as per the 2^-ΔΔCT method.

### 2.9 Western blotting

The Western blot assay was conducted following established protocols (Gao et al., 2021). In brief, MC3T3-E1 cells were incubated with the leaching liquor of IONP-CMs for 3 days. Then, the cells were harvested and lysed employing radioimmunoprecipitation assay lysis buffer, to which 1 mM phenylmethylsulfonyl fluoride was added. The total intracellular protein was extracted, subjected to separation through 10% SDS-PAGE, and subsequently transferred onto PVDF membranes via electroblotting. In order to reduce the occurrence of non-specific binding, the membranes underwent blocking with 5% skimmed milk and were thereafter incubated overnight with primary antibodies at 4°C. Following this, the membranes were washed with Tris-buffered saline and Tween 20 three times. Subsequently, the membranes were exposed to secondary antibodies for another 1 h. Ultimately, the Western blots were visualized using an ECL plus Western blotting detection system, enabling the visualization of the protein bands.

### 2.10 Animal experiments

Male New Zealand rabbits, with an average weight of 4 kg, were purchased from the Animal Study Committee of Southeast University (China). All experimental protocols in this study were approved by the Committee of Zhongda Hospital of Southeast University (No. 20210901001). All surgical procedures and animal care protocols were performed following the Guide for the Care and Use of Laboratory Animals published by the U.S. National Institutes of Health. The animals were randomly categorized into different treatment groups.

### 2.11 Radial defect model

Male New Zealand rabbits were narcotized by intravenous injection of propofol and lidocaine. Then, a 10-mm defect was made in their midshaft of radius, and subsequently the IONP-CM samples were implanted as the bone repair scaffold, where neat CM was used as control. The blank group did not undergo any surgical procedures. Administration of cephalosporin was necessary to prevent potential infections. Subsequently, at the intervals of 3, 6, and 9 weeks following the surgeries, the rabbits were humanely euthanized and their radiuses were carefully extracted and collected for subsequent assessment of bone regeneration.

### 2.12 Micro-computed tomography assessment

The harvested rabbit radius samples underwent testing using the vivaCT 80 system (V6.5-3 Scanco Medical, Bruttisellen, Switzerland). The imaging parameters employed were as follows: a voxel size of 9 μm, a scanning voltage of 55 keV, and a scanning current of 145 μA. Subsequently, a range of trabecular indices were computed, including bone mineral density (BMD), bone volume/total volume (BV/TV), trabecular number (Tb.N), trabecular separation (Tb.Sp), and trabecular thickness (Tb.Th). To further evaluate bone regeneration, the micro-CT images were subjected to additional analysis employing the watershed and AdaBoost algorithms. This additional assessment aimed to provide a comprehensive understanding of the bone regeneration process based on the acquired micro-CT images.

### 2.13 Histological analysis

The harvested rabbit radius samples were subjected to a series of preparatory steps. They were initially rinsed three times with PBS to ensure the removal of residual contaminants. Following this, the samples were fixed using formalin and maintained at a temperature of 4°C for 24 h. Subsequently, a decalcification process was carried out utilizing 15% ethylenediaminetetraacetic acid solution, spanning 28 days. Following successful decalcification, the radius samples were again rinsed repeatedly with PBS. For further processing, the decalcified radius samples were embedded within paraffin material, facilitating subsequent slicing into 5-μm-thick sections. These as-prepared sections underwent staining using hematoxylin and eosin (H&E) dyes. The stained sections were then subjected to microscopic observation using a microscope equipped with a CCD camera.

### 2.14 Statistical analysis

Statistical comparisons between the various groups were conducted using a one-way analysis of variance (ANOVA) followed by a Tukey *post-hoc* analysis. A significance level of *p* < 0.05 was considered indicative of statistically significant differences between the groups. The entire statistical analysis was executed using GraphPad Prism 8, a widely used software tool for statistical and graphical analysis.

## 3 Results and discussion

### 3.1 Preparation and characterization of IONPs

**FIGURE 1:**
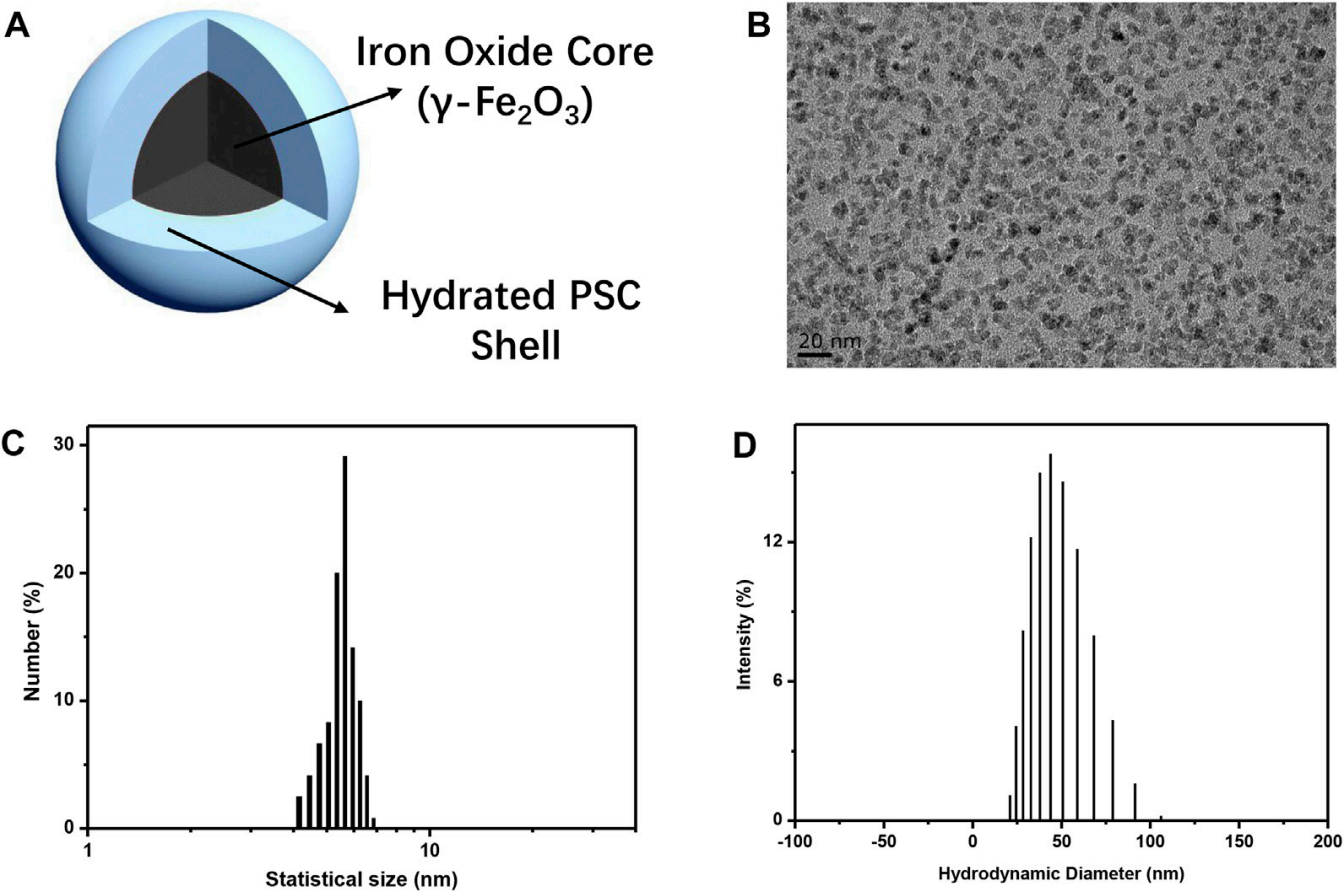
Preparation and characterization of IONPs. **(A)** Schematic of PSC-coated IONPs. **(B)** TEM image of as-prepared IONPs. **(C)** Statistical size distribution of IONPs. **(D)** Hydrodynamic diameter distribution of as-prepared IONPs.

The primary objective of this study was to create biocompatible iron oxide nanoparticles (IONPs) and subsequently integrate them into a composite material. This composite material was intended to serve as a scaffold, with the ultimate aim of facilitating the regeneration of bone tissue. Hence, IONPs were prepared using the classical chemical co-precipitation method reported in previous studies, where PSC was employed as a stabilizer to avoid the aggregation of nanoparticles and guarantee their biocompatibility (Wang et al., 2020). The nanoparticles that were synthesized in this study exhibited distinctive core–shell architecture, as depicted in Figure 1A. This hierarchical structure comprised a central core composed of γ-Fe_2_O_3_, encapsulated within a surrounding shell made of a polymeric self-crosslinking (PSC) material. The topography of IONPs was observed by TEM, as depicted in Figure 1B, revealing that the nanoparticles were spherical and of high monodispersity. High-resolution transmission electron microscopy (HRTEM) of IONPs (Supplementary Figure S1) showed the lattice of nanoparticles, indicating them as being crystallized. The statistical mean size of IONPs according to the TEM image was 5.54 nm (Figure 1C). Furthermore, their hydrodynamic diameters were determined using dynamic light scattering. The analysis revealed a mean hydrodynamic diameter of 45.7 nm, as illustrated in Figure 1D, the value of which exceeded their average physical size due to the presence of the PSC molecular chains and the hydrated shell surrounding the nanoparticles. The obtained polydispersity index for the synthesized IONPs was measured at 0.239, underscoring their exceptional dispersibility. Additionally, the colloidal IONPs exhibited a negatively charged zeta (ζ) potential with the value −22.5 mV due to the carboxyl group of PSCs on the surface of IONPs, confirming their surface charge characteristics. The preparation process of PSC-coated IONPs exactly followed that of the only inorganic nanodrug approved by the FDA for iron deficiency anemia treatment (brand name: ferumoxytol). Our as-prepared IONPs (brand name: Ruicun) were also approved by the National Medical Products Administration for iron-supplementary and magnetic resonance imaging (MRI) contrast agents, and we believed that they were biocompatible nanoparticles and could be used as osteogenic agents to incorporate into the CM for further bone regeneration evaluation.

### 3.2 Surface topography of IONP-CMs

**FIGURE 2:**
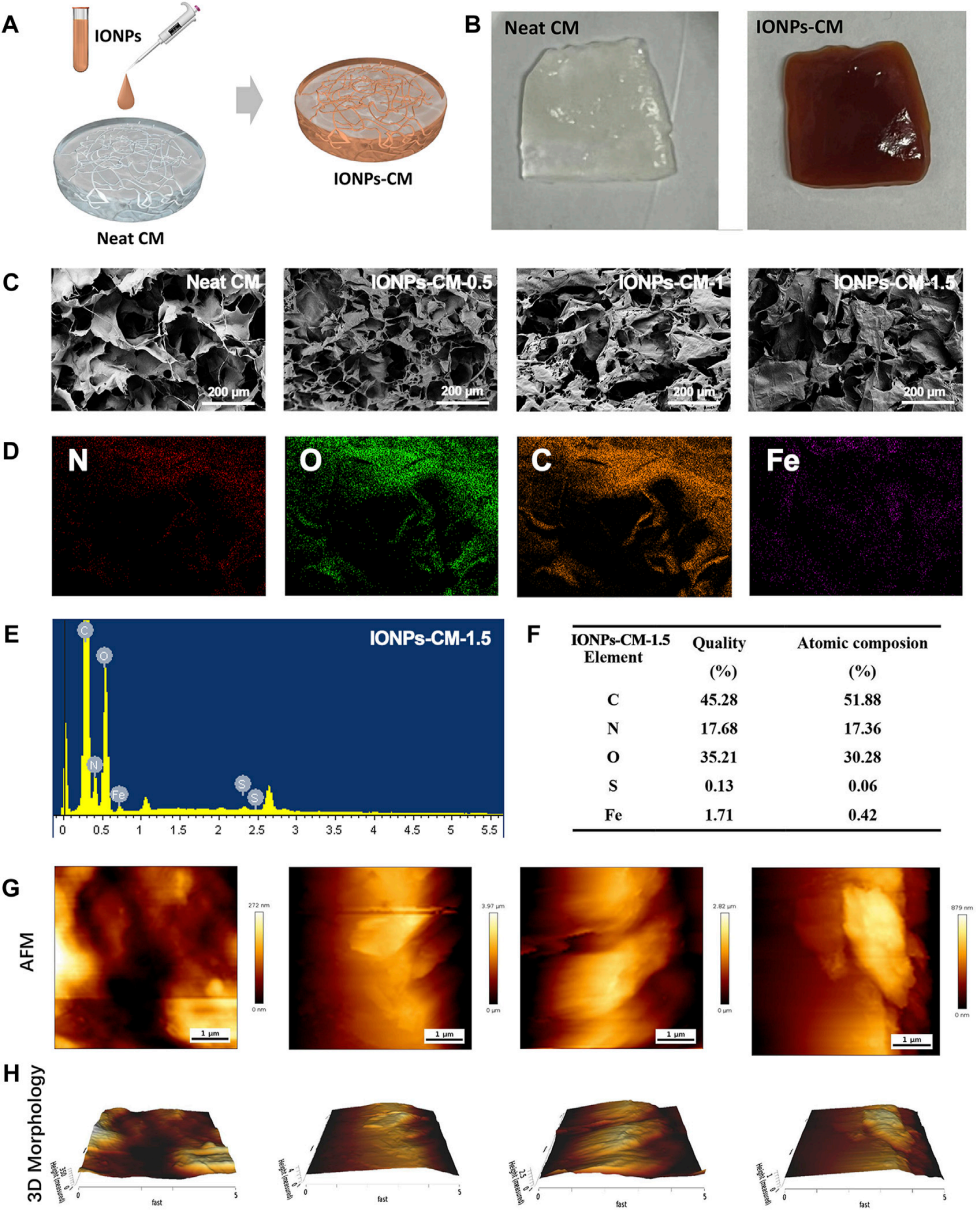
Surface topography of IONP-CM. **(A)** Schematic of preparation of IONP-CM. **(B)** Photographs of the neat CM and IONP-CM, respectively. **(C)** SEM images of the neat CM, IONP-CM-0.5, IONP-CM-1, and IONP-CM-1.5. **(D)** Elemental mapping (C, N, O, and Fe) of IONP-CM-1.5 according to the SEM image. **(E)** EDS spectra and **(F)** semi-quantitative determination of quality and atomic composition of IONP-CM-1.5. **(G)** Typical AFM surface images and their corresponding **(H)** 3D morphologies of IONP-CMs.

IONP-CM was prepared by simply mixing the IONP solution with neat CM, the process of which is visually depicted in Figure 2A. Photographs of the neat CM and as-prepared IONP-CMs are shown in Figure 2B, in which the color of IONP-CMs is brown due to the incorporation of IONPs. The as-prepared IONP-CMs were used as smart scaffolds for bone tissue regeneration assessment. Hence, three types of IONP-CMs (IONP-CM-0.5, IONP-CM-1, and IONP-CM-1.5) were prepared by incorporating various concentrations of IONPs (iron content: 0.5%, 1%, and 1.5%) into the pure CM. The surface topography of bone repair scaffolds stands as a pivotal determinant for effective bone tissue regeneration. This significance arises from its capability to influence various cellular functions, including cell proliferation and differentiation (Huang et al., 2021). The morphology of the neat CM, IONP-CM-0.5, IONP-CM-1, and IONP-CM-1.5 was first characterized. As shown in Figure 2C, the SEM images revealed that neat CM possessed a porous structure. As expected, the IONP-incorporated CMs still possessed porous nanostructures, but their morphological surface turned rough compared with that of neat CM, which could be attributed to the interaction between the functional groups of IONPs and neat CM. The elemental mapping images of IONP-CM-1.5 measured using EDS (Figure 2D) showed N, O, C, and Fe in a uniformly distributed state, indicating that IONPs were homogeneously distributed without apparent aggregation. Furthermore, the elemental quality and atomic composition were quantitatively calculated. The results are shown in Figures 2E, F, demonstrating that the elemental quality (atomic composition) of C, N, O, and Fe was 45.28% (51.88%), 17.68% (17.36%), 35.21% (30.28%), and 1.71% (0.42%), respectively. Furthermore, we harnessed *in situ* high-resolution AFM technology to examine both the planar view (Figure 2G) and three-dimensional (3D) morphology (Figure 2H) of the samples. Intriguingly, the introduction of IONPs exhibited a minimal impact on the inherent surface nanostructural attributes of the pristine CM. Moreover, based on the AFM findings, we calculated Young’s modulus of the IONP-CM composite. The value of Young’s modulus for the neat CM was 2.81 MPa. The value was 4.41 MPa for IONP-CM-0.5, 5.17 MPa for IONP-CM-1, and 6.27 MPa for IONP-CM-1.5. The increased Young’s modulus of the CM might be attributed to the addition of IONPs.

### 3.3 Physicochemical properties of IONP-CM

**FIGURE 3:**
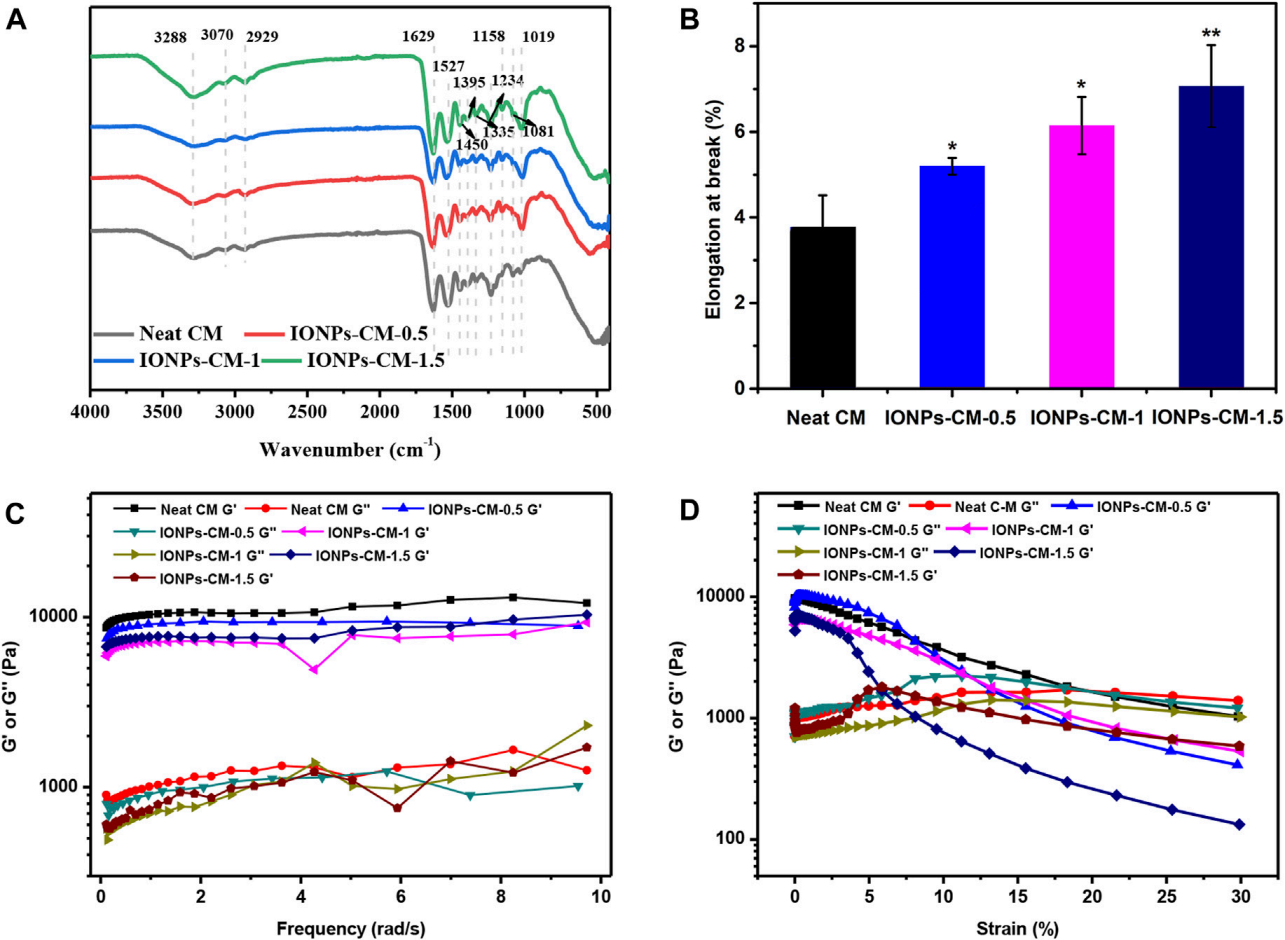
Physicochemical properties of IONP-CMs. **(A)** FTIR spectra and **(B)** elongation at break of the neat CM, IONP-CM-0.5, IONP-CM-1, and IONP-CM-1.5. **(C, D)** Dynamic mechanical properties of various samples were characterized through both frequency–sweep testing (ranging from 0.1 to 60 rad/s, with a 1% strain) and strain–sweep testing (ranging from 0.1% to 10% strain, at a frequency of 6.28 rad/s) at room temperature. The asterisk indicates statistically significant differences between the control and experimental groups (^∗^ *p* < 0.05; ^∗∗^ *p* < 0.01).

The surface chemical structure of IONP-CMs was characterized using FTIR. As shown in Figure 3A, all spectra of IONP-CMs demonstrated the typical absorption bands as the neat CM (Gui-Long et al., 2003), indicating that the incorporation of IONPs barely affected the chemical structure of the CM. The absorption peaks at 1,234, 1,335, 1,395, and 1,450 cm^-1^ in FTIR spectra were associated with amide III, in-plane bending vibration of CH_2_, shear in-plane bending vibration of CH_3,_ and anti-symmetric in-plane bending vibration of CH_2_, respectively. These results indicated that the triple-helical structure of collagen was not markedly changed after the incorporation of IONPs. The peaks at 3,288, 3,070, and 1,527 cm^-1^ were attributed to amide A, amide B, and amide II, respectively, indicating many hydrogen bonds in the IONP-CM. This result was similar to the findings of Pieper et al. (1999). Furthermore, the mechanical properties of biomaterials used as scaffolds are of great importance for bone tissue regeneration because scaffolds with proper mechanical properties may provide an appropriate microenvironment for multiple cell functions, including cell growth, cell adhesion, and cell differentiation. The tensile performances of the as-prepared IONP-CMs were detected. The elongation at break is shown in Figure 3B, revealing that the neat CM possessed the lowest tensile strain of 3.77%. However, the IONP-CM demonstrated an increased tensile strain of 5.19% for IONP-CM-0.5, 6.14% for IONP-CM-1, and 7.06% for IONP-CM-1.5. The increased tensile strain might be attributed to the addition of various amounts of IONPs. Moreover, we conducted comprehensive static and dynamic rheological analyses on the IONP-CM composite using a rheometer. The outcomes from the frequency–sweep test (Figure 3C) demonstrated that the storage modulus (G′) notably exceeded the loss modulus (G″), underscoring the mechanical robustness of the composite. The results of the strain–sweep test (Figure 3D) demonstrated a strain at yield (the cross point of *G*′ and *G*″) of 19.76% for the neat CM, 11.97% for IONP-CM-0.5, 15.59% for IONP-CM-1, and 5.73% for IONP-CM-1.5, which implied the transition of the network of gel to a liquid state. Furthermore, the cumulative release of iron (Fe) content from the IONP-CM (iron oxide nanoparticle-coated cell culture medium) was quantified in PBS during a 7-day incubation period, employing inductively coupled plasma mass spectrometry (ICP-MS) analysis. As illustrated in Supplementary Figure S2, the obtained data exhibited a time-dependent increase in the release of Fe ions, and the accumulated release quantity was notably enhanced upon the addition of IONPs. These results indicated that the IONP-CM with remarkable physicochemical properties could be considered a repair scaffold for bone tissue regeneration.

### 3.4 Cell viability and affinity of MC3T3-E1 cells toward IONP-CMs

**FIGURE 4:**
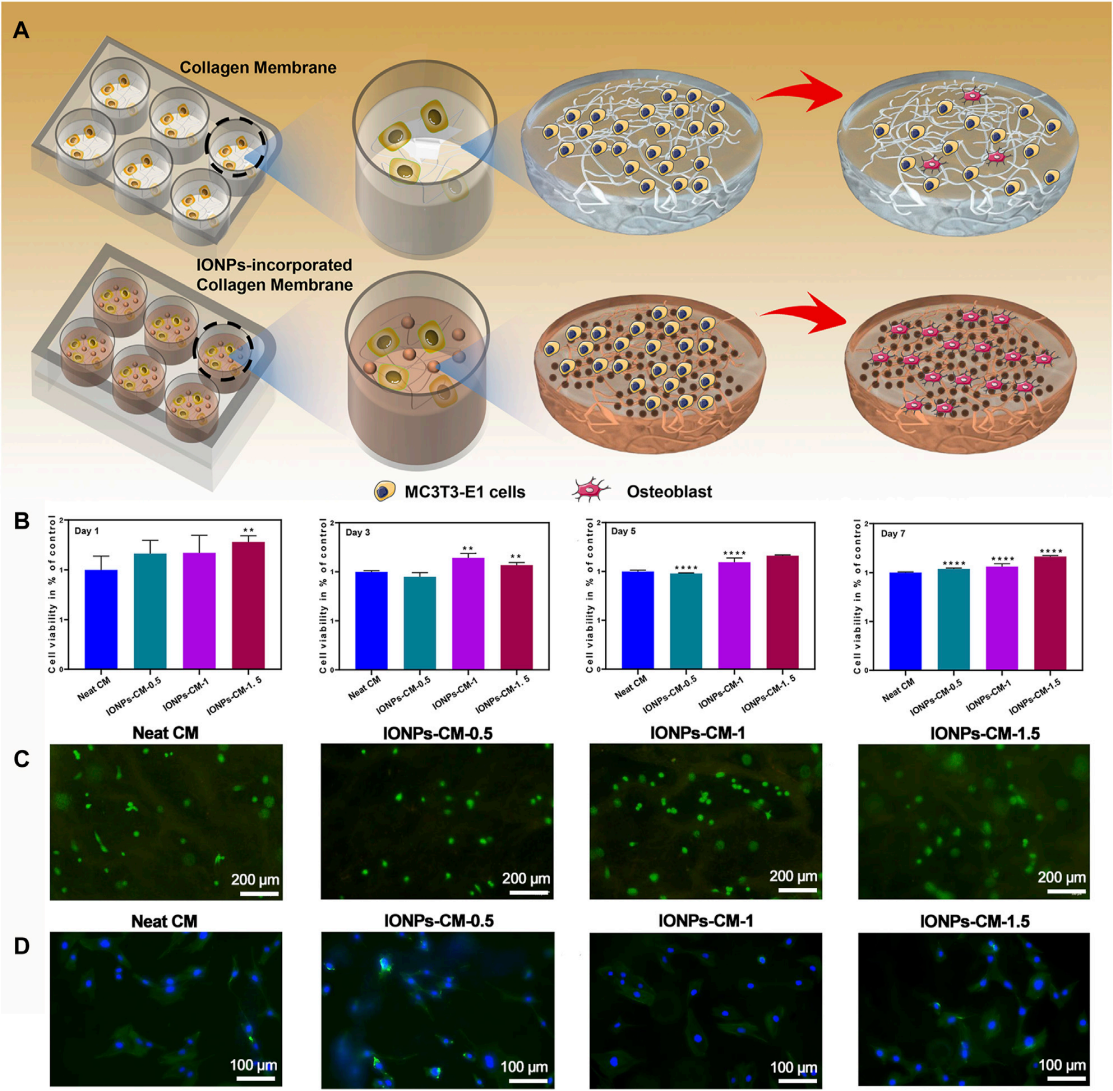
Biocompatibility of IONP-CMs. **(A)** Schematic representation for cellular viability and osteogenic differentiation of IONP-CMs. **(B)** Cell viability of MC3T3-E1 cells after treatment with the neat CM, IONP-CM-0.5, IONP-CM-1, and IONP-CM-1.5 for 1, 3, 5, and 7 days, respectively. **(C)** Illustrative visual of live/dead staining for MC3T3-E1 cells following 24 h of exposure to the as-prepared CM, where green and red fluorescence represent live and dead cells, respectively. **(D)** Representative scan of phalloidin/DAPI staining of MC3T3-E1 cells after treatment with the aforementioned CM for 24 h. The staining highlights the cellular cytoskeleton in red, achieved through rhodamine phalloidin, and the nuclei in blue, achieved through DAPI staining. The asterisk indicates statistically significant differences between the control and experimental groups (^∗∗∗∗^ *p* < 0.001).

Biomaterials as bone repair scaffolds should show no cytotoxicity to the body, facilitating cell adhesion and proliferation for bone tissue regeneration. Consequently, we proceeded to assess the *in vitro* biocompatibility of the IONP-CM composite by employing MC3T3-E1 cells as the cellular model. The schematic diagram is shown in Figure 4A. Following the cultivation of MC3T3-E1 cells for 1, 3, 5, and 7 days, it was observed that both neat CM and IONP-CMs demonstrated negligible cytotoxic effects, with average cell viability surpassing 95%. MC3T3-E1 cells cultured with IONP-CMs demonstrated a higher cell proliferation rate than the neat CM after 5 and 7 days of co-incubation, indicating that the addition of IONPs promoted cell proliferation (Figure 4B). Furthermore, live/dead staining (Figure 4C) was used to assess the biocompatibility of IONP-CMs visually. Almost all the cells showed green fluorescence, indicating that the cells were alive. In general, numerous investigations confirmed that IONPs could facilitate cell proliferation *in vitro* (Lu et al., 2021). In addition, cell adhesion was assessed using phalloidin/DAPI staining. As depicted in Figure 4D, after 24 h of co-incubation, the results demonstrated that the neat CM offered favorable adhesion sites for cell survival, which spread out and maintained their spindle-shaped morphology. As expected, adding IONPs into CM did not affect cell adhesion. A similar trend could be seen after 48 h of co-incubation (Supplementary Figure S3). These results indicated that IONP-CMs had significant biocompatibility and could provide a proper environment for cell adherence and proliferation.

### 3.5 Osteogenic differentiation of MC3T3-E1 cells toward IONP-CMs

**FIGURE 5:**
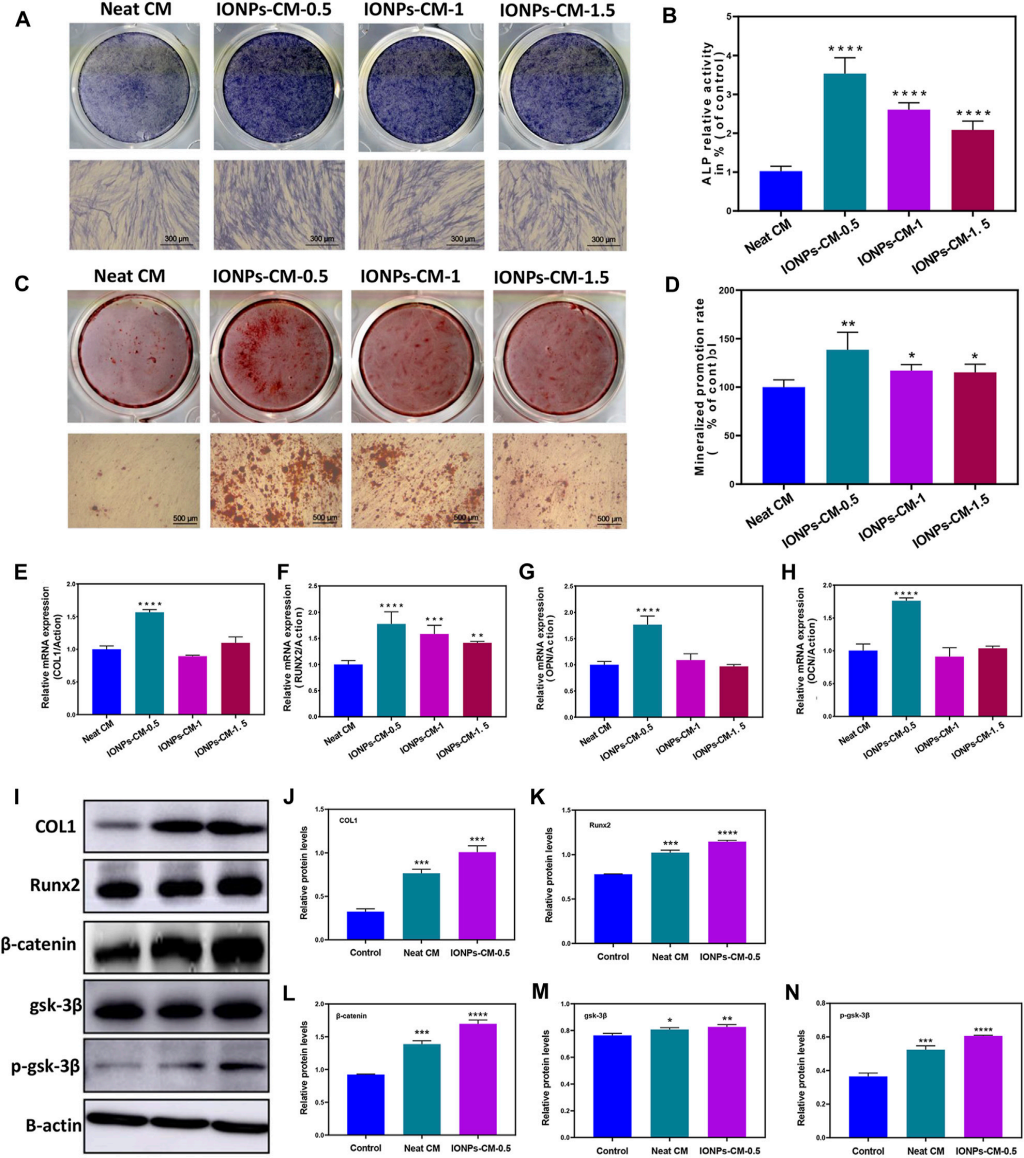
Assessment of osteogenic differentiation of IONP-CMs. **(A)** ALP staining and **(B)** ALP activity assessment of MC3T3-E1 cells following exposure to leaching liquors from the neat CM, IONP-CM-0.5, IONP-CM-1, and IONP-CM-1.5 for 7 days. **(C)** ARS staining and **(D)** determination of mineralization promotion rate of MC3T3-E1 cells post exposure to leaching liquors from the aforementioned CMs for 21 days. **(E–H)** RT-PCR analysis of COL1, Runx2, OPN, and OCN gene expressions in MC3T3-E1 cells following treatment with leaching liquors from the aforementioned CMs for 7 days. **(I)** Western blot analysis and **(J–N)** assessment of relative protein expressions of COL1, Runx2, β-catenin, GSK-3β, and p-GSK-3β in MC3T3-E1 cells post treatment with leaching liquors from the aforementioned CMs for 7 days. The asterisk indicates statistically significant differences between the control and experimental groups (^∗^ *p* < 0.05; ^∗∗^ *p* < 0.01; ^∗∗∗^ *p* < 0.005; ^∗∗∗∗^ *p* < 0.001).

Proper scaffolds used in bone tissue regeneration may have superior biological activities, such as osteo-conductivity and osteo-inductivity, toward natural bone tissue to facilitate the osteogenic differentiation of biological cells and avoid the formation of fibrous connective tissue (Xia et al., 2018). Endowing current biomaterials with excellent osteogenic bioactivities is of great significance when used as bone repair scaffolds. Specific markers, including ALP activities, formation of mineralized nodules, and osteogenesis-related mRNA expression, were involved in the osteogenic differentiation of biological cells (Zhang et al., 2017). Among these specific markers, ALP is considered an early specific marker that can reveal the osteogenic bioactivity of osteoblasts. Hence, ALP activity in MC3T3-E1 cells treated with the leaching liquor of the neat CM and IONP-CMs for 7 days was first investigated using ALP staining and ALP activity assay. As demonstrated in Figure 5A, the neat CM exhibited a relatively lower ALP activity than the IONP-incorporated CM, and IONP-CM-0.5 showed the highest ALP activity. The ALP activity assay demonstrated the same tendency (Figure 5B), with an approximately 350% increase in IONP-CM-0.5, 260% increase in IONP-CM-1, and 209% increase in IONP-CM-1.5. Based on the fact that IONPs were reported to have excellent osteogenic bioactivities, the aforementioned results might be attributed to the incorporation of IONPs. Furthermore, we detected the mineralized nodule formation of MC3T3-E1 cells treated with the leaching liquor of IONP-CM for 21 days using ARS staining. As demonstrated in Figure 5C, the addition of IONPs could endow the neat CM with improved formation of mineralized nodules, and the mineral deposition of MC3T3-E1 cells cultured with IONP-CM-0.5 was markedly higher than that of IONP-CM-1 and IONP-CM-1.5. Furthermore, the semi-quantified mineralized calcium deposition of MC3T3-E1 cells (Figure 5D) was detected by dissolving the ARS deposits and eluting their absorbance (Zhu et al., 2020). An increased amount of mineralized matrix was observed after the incorporation of IONPs, which was consistent with the ARS staining results. In addition, several osteogenesis-related genes, such as COL1, Runx2, OPN, and OCN, were upregulated during osteogenic differentiation (Kuterbekov et al., 2020). Hence, we quantitatively detected the expression of these mRNAs using real-time PCR (RT-PCR). As shown in Figures 5E–H, incorporating IONPs endowed the neat CM with enhanced expression of osteogenesis-related genes, and IONP-CM-0.5 demonstrated the highest upregulation of these mRNAs. We also performed Western blot to evaluate the protein expression level of osteogenesis-related genes (COL1 and Runx2) to further verify the osteogenic activities of IONP-CMs. The results are shown in Figures 5I–K. The aforementioned genes were markedly upregulated in MC3T3-E1 cells treated with IONP-CM-0.5, which was consistent with the mRNA data. However, the effects of bone repair scaffolds on the osteogenic differentiation of biological cells are complex. Numerous studies have highlighted the role of IONPs in promoting osteogenic differentiation and bone regeneration, which are attributed to their ability to activate critical signaling pathways, including the Wnt/β-catenin and ERK/MAPK pathways (Xia et al., 2019). Our findings revealed a significant upregulation of the Runx2 gene at both the mRNA and protein levels in response to IONP-CM treatment. Given that Runx2 is a key gene associated with the Wnt/β-catenin signaling pathway, we postulated that this pathway might be pivotal in mediating the osteogenic effects induced by IONP-CMs. To further substantiate this hypothesis, we investigated the specific markers linked to the Wnt/β-catenin pathway (β-catenin, GSK-3β, and p-GSK-3β) employing Western blotting. The Western blot membranes (Figure 5I) and the corresponding relative protein levels (Figure 5L–N) exhibited elevated expression of these markers in MC3T3-E1 cells treated with IONP-CMs in comparison to the control and neat CM groups. This outcome lends preliminary support to the notion that the Wnt/β-catenin signaling pathway likely plays a pivotal role in mediating the osteogenic effects elicited by IONP-CMs.

### 3.6 Mechanism of IONP-CM-induced osteogenic differentiation

**FIGURE 6:**
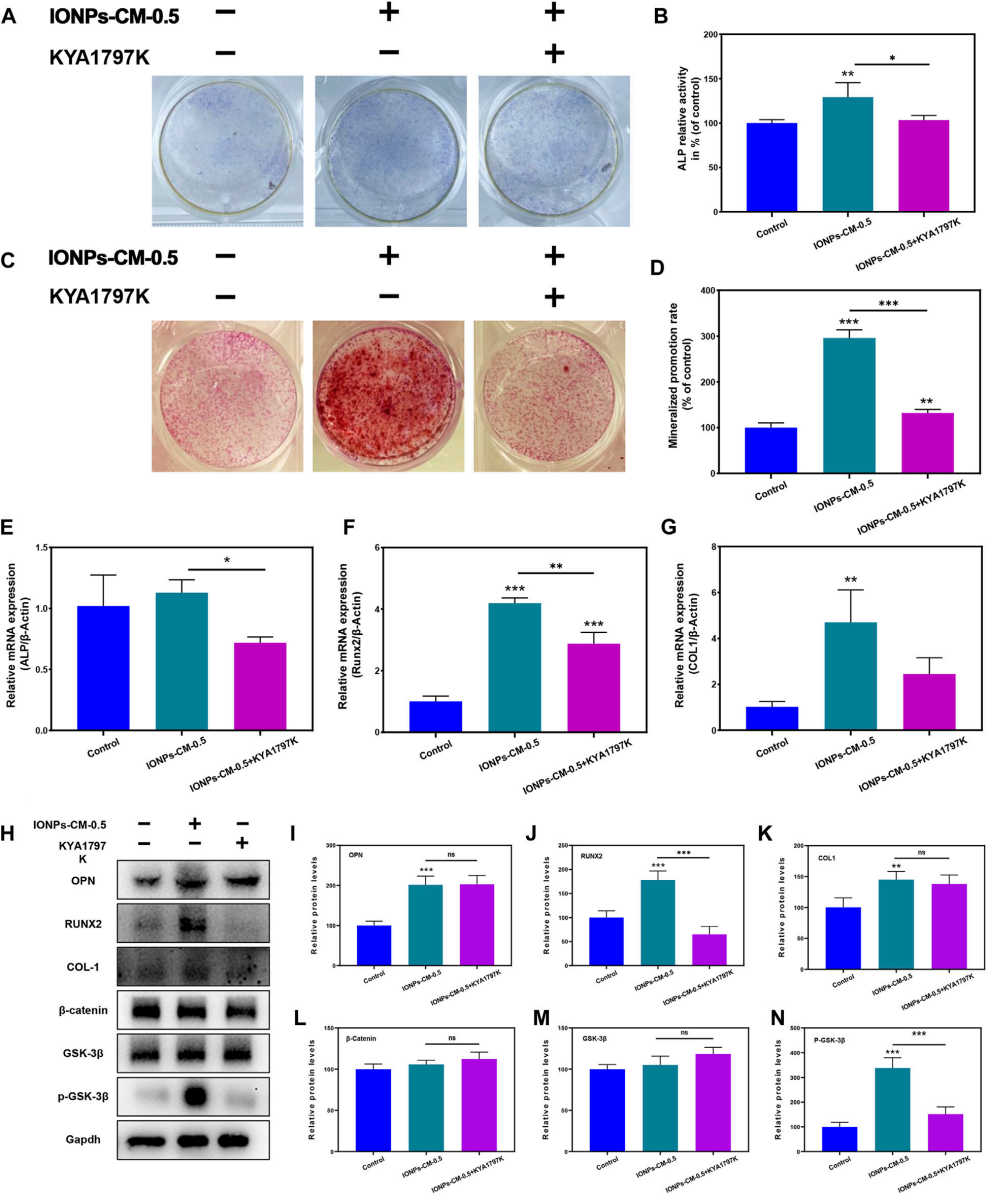
Mechanism of IONP-CM-induced osteogenic differentiation. **(A)** ALP staining and **(B)** ALP activity level of MC3T3-E1 cells after treatment with IONP-CM-0.5 alone or combined with KYA1797K for 7 days. **(C)** ARS staining and **(D)** mineralized promotion rate of MC3T3-E1 cells treated with IONP-CM-1.5 alone or combined with KYA1797K for 21 days. **(E–G)** RT-PCR analysis of ALP, COL1, and Runx2 expression in MC3T3-E1 cells after treatment with IONP-CM-1.5 alone or combined with KYA1797K for 7 days. **(I)** Western blot analysis and **(J–N)** relative OPN, COL1, Runx2, β-catenin, and p-GSK-3β protein expression in MC3T3-E1 cells after treatment with IONP-CM-1.5 alone or combined with KYA1797K for 7 days. The asterisk indicates statistically significant differences between the control and experimental groups (^∗^ *p* < 0.05; ^∗∗^ *p* < 0.01; ^∗∗∗^ *p* < 0.005).

Mechanistic investigations were performed to validate whether the Wnt/β-catenin signaling pathway affected IONP-CM-induced osteogenic differentiation using a specific Wnt/β-catenin signaling inhibitor, KYA1797K, which could decrease the expression of β-catenin and subsequently block the signal transduction pathway of Wnt/β-catenin (Zhang et al., 2021). The ALP staining (Figure 6A) and ALP activity level (Figure 6B) showed that adding KYA1797K significantly reversed IONP-CM-0.5-induced osteogenic differentiation of MC3T3-E1 cells. Similarly, the enhanced ARS staining (Figure 6C) and quantified mineralized nodules (Figure 6D) induced by IONP-CM-0.5 were markedly blocked after KYA1797K treatment. In addition, we detected the osteogenesis-related gene expression in MC3T3-E1 cells treated with IONP-CM-0.5 alone or combined with KYA1797K using RT-PCR. As demonstrated in Figures 6E–G, the expression of these osteogenesis-related genes (ALP, COL1, and Runx2) was significantly upregulated after IONP-CM-0.5 treatment, while the addition of KYA1797K markedly reversed this IONP-CM-0.5-induced osteogenesis. Furthermore, the Western blot analysis of OPN, Runx2, and COL1 revealed the same tendency as the RT-PCR data (Figures 6H–K). β-catenin and p-GSK-3β (typical proteins involved in the Wnt/β-catenin signaling pathway) were also upregulated after the IONP-CM-0.5 treatment and subsequently blocked by KYA1797K (Figure 6L–N). The outcomes derived from these investigations revealed a noteworthy insight that the introduction of the Wnt/β-catenin signaling inhibitor KYA1797K led to the reversal of IONP-CM-0.5-induced osteogenic differentiation in MC3T3-E1 cells, evident at both the mRNA and protein levels. As a result, we are inclined to propose that IONP-CM-0.5 may indeed play a role in promoting the osteogenic differentiation of MC3T3-E1 cells by means of activating the Wnt/β-catenin signaling pathway.

### 3.7 Assessment of *in vivo* bone regeneration

**FIGURE 7:**
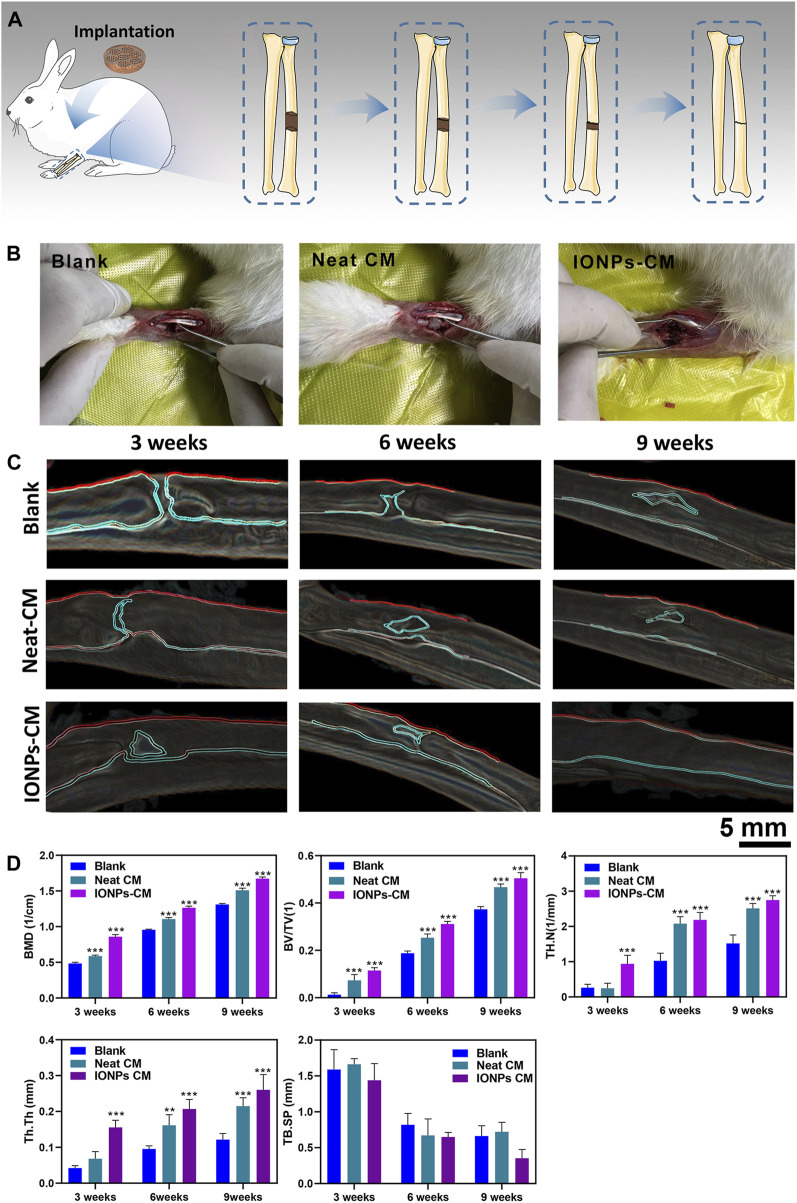
Assessment of *in vivo* bone regeneration. **(A)** Schematic diagram of the experimental procedure. **(B)** Photographs of the bone defect area alone or implanted with the neat CM or IONP-CM. **(C)** Micro-CT images of the midshaft of the radius of rabbits; the defect areas were recognized using the watershed and AdaBoost algorithms. **(D)** Quantitative analysis of BMD, BV/TV, and bone trabecula parameters (TB·N, TB. Sp, and TB.Th). The asterisk indicates statistically significant differences between the control and experimental groups (^∗∗^ *p* < 0.01; ^∗∗∗^ *p* < 0.005).

**FIGURE 8:**
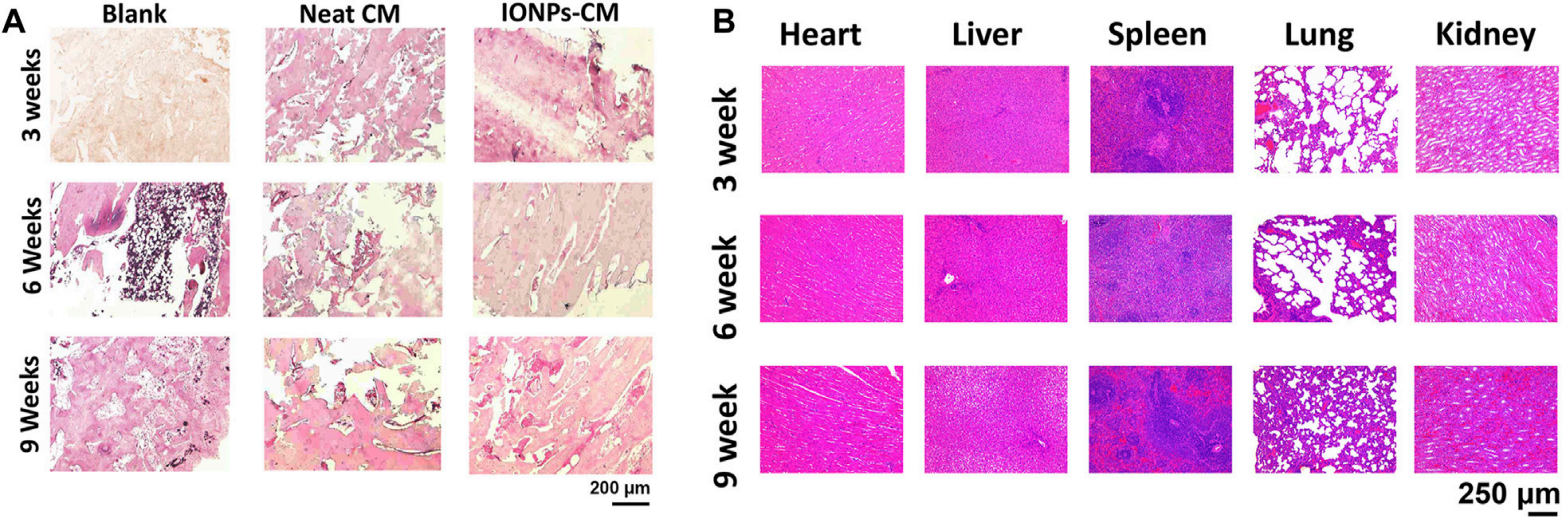
Histological analysis. **(A)** Histological staining images of the defect areas of the midshaft of the radius implanted with the neat CM and IONP-CM-0.5. **(B)** H&E staining images of major organs collected from rabbits administered IONP-CM-0.5 at 3, 6, and 9 weeks after implantation.

Based on the *in vitro* investigations, IONP-CM-0.5 demonstrated the most optimal performance for facilitating the osteogenic differentiation of biological cells. Therefore, IONP-CM-0.5 was selected for the *in vivo* assessment of bone regeneration performance using the midshaft of rabbits with radial defect as the model (Figure 7A). IONP-CM-0.5 was directly implanted into the defect area of the midshaft of the radius (Figure 7B), where the neat CM was implanted as the control. The femurs of rabbits were harvested to evaluate bone regeneration 3, 6, and 9 weeks after implantation. Based on the sectional images of the midshaft of the radius observed by CT, we first employed the watershed algorithm to obtain the area of the defect using the commercial software MATLAB 7.0. Subsequently, image segmentation was accomplished using the AdaBoost algorithm based on the textural features and geometrical morphology of the bone defect area (Baig et al., 2017). As shown in Figure 7C, the defect site was still discontinuous after 3 weeks in the blank and control groups; however, some connected sites were observed in the IONP-CM-0.5 group. Less defect area was observed in the IONP-CM-0.5 group compared with the blank and control groups, indicating that the IONP-CM accelerated bone regeneration. We calculated the bone structure parameters, including BMD, BV/TV, Tb.N, Tb.Th, and Tb. Sp, in rabbits administered the neat CM and IONP-CM-0.5 at 3, 6, and 9 weeks after implantation based on the CT images to quantitatively assess their promoting performance. The results are demonstrated in Figure 7D, revealing significant increments in BMD, BV/TV, Tb.N, and Tb.Th in the IONP-CM-0.5 group compared with the neat CM and blank groups. However, the value of Tb. Sp in the defect area treated with IONP-CM-0.5 was relatively lower than that in the neat CM and blank groups. These results indicated that the IONP-CM accelerated *in vivo* bone regeneration. Furthermore, the regenerated bone tissue in the defects treated with the neat CM and IONP-CM-0.5 at 3, 6, and 9 weeks after the surgery was histologically examined using H&E staining. As illustrated in Figure 8A, fibrous tissue was observed in the bone defect area treated with IONP-CM-0.5 for 3 weeks. However, obvious intervals between tissue lesions and fibrous tissue were found in the neat CM group. After 9 weeks of treatment, only a few capillaries and fibrous tissue were observed in the blank group, and a small amount of bone matrix was formed in the neat CM group. The mature bone matrix and osteocytes with good cellular morphology were found in the IONP-CM-0.5 group. These findings indicated that IONP-CMs could effectively promote the repair of bone defects. Finally, the *in vivo* biocompatibility of the IONP-CMs was assessed by histological examination. As demonstrated in Figure 8B, H&E staining of the major organs, including the heart, liver, spleen, lung, and kidney, of rabbits administered IONP-CM-0.5 did not show obvious inflammation and noticeable pathological characteristics, indicating that the IONP-CM was nontoxic to the body. Because the IONP-CM demonstrated remarkable physicochemical properties and excellent osteogenic bioactivities, we believed that it could be used as a promising scaffold for bone tissue regeneration.

## Data Availability

The original contributions presented in the study are included in the article/Supplementary Material; further inquiries can be directed to the corresponding author.
