# Supplementary material for: Endowing improved osteogenic activities with collagen membrane by incorporating biocompatible iron oxide nanoparticles
Source: Front Bioeng Biotechnol. 2023 Oct 12;11:1259904. doi: 10.3389/fbioe.2023.1259904 (PMC10601650; doi:10.3389/fbioe.2023.1259904)
Supplement: Supplementary file 1 [file DataSheet1.docx]

**Supporting information**

Endowing Improved Osteogenic Activities with Collagen Membrane by Incorporating Biocompatible Iron Oxide Nanoparticles

*
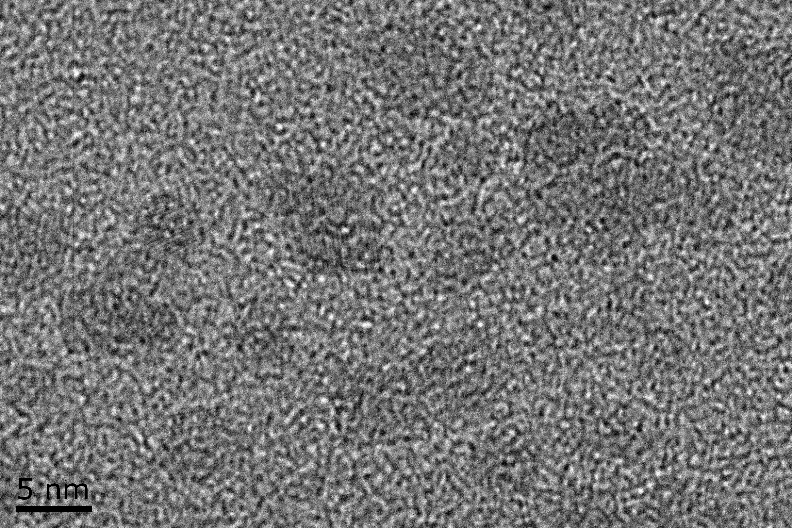
*

**Figure S1**. High resolution transmission electron microscopy image of as-prepared IONPs.





**Figure S2.** IONPs release in PBS buffer from 1 to 7 d of CM scaffolds.


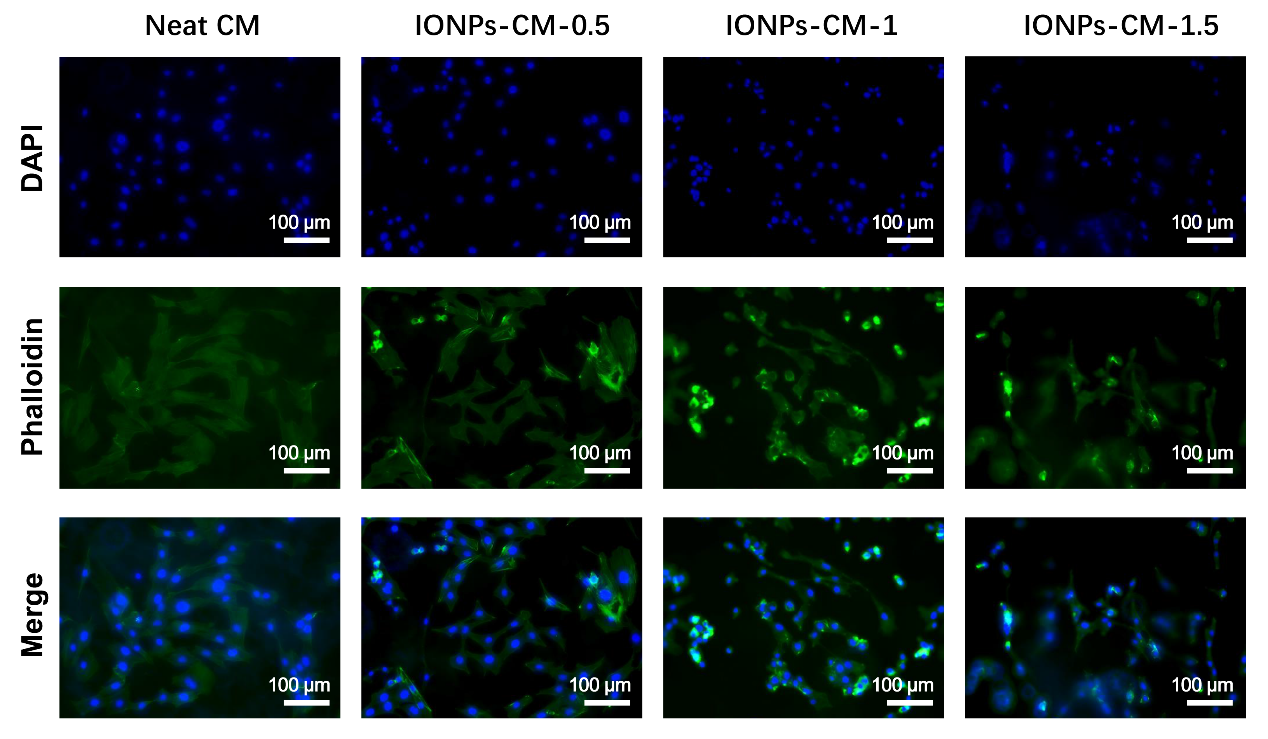


**Figure S3.** Fluorescent staining of MC3T3-E1 cells cultured with IONPs-CM for 48 h. Red, cytoskeleton stained by rhodamine phalloidin; Blue, nuclei stained by DAPI.
